# Correction to “Dermoscopy and reflectance confocal microscopy‐augmented characterization of pigmented micro‐basal cell carcinoma (less than 2 mm diameter).”

**DOI:** 10.1111/srt.70306

**Published:** 2026-02-10

E. Foltz, J. Ludzik, A.L. Becker, E. Latour, C. Lee, and A. Witkowski, “Dermoscopy and Reflectance Confocal Microscopy‑Augmented Characterization of Pigmented Micro‑Basal Cell Carcinoma (Less Than 2 mm Diameter)” *Skin Research and Technology* 29 no. 1 2023: e13250, https://doi.org/10.1111/srt.13250

In the published version, the institutional affiliation of author Joanna Łudzik was incomplete. Only the Oregon Health & Science University affiliation was listed. The correct affiliations should read:

1. Oregon Health & Science University, Department of Dermatology, Portland, USA

2. Department of Bioinformatics and Telemedicine, Jagiellonian University Medical College, Kraków, Poland.

We apologize for this error.

